# Heterologous Expression of Secreted Bacterial BPP and HAP Phytases in Plants Stimulates *Arabidopsis thaliana* Growth on Phytate

**DOI:** 10.3389/fpls.2018.00186

**Published:** 2018-02-20

**Authors:** Lia R. Valeeva, Chuluuntsetseg Nyamsuren, Margarita R. Sharipova, Eugene V. Shakirov

**Affiliations:** ^1^Institute of Fundamental Medicine and Biology, Kazan (Volga Region) Federal University, Kazan, Russia; ^2^Department of Integrative Biology, The University of Texas at Austin, Austin, TX, United States

**Keywords:** phosphorus, phytate, phytase, transgenic plant, soil bacteria, Arabidopsis

## Abstract

Phytases are specialized phosphatases capable of releasing inorganic phosphate from *myo*-inositol hexakisphosphate (phytate), which is highly abundant in many soils. As inorganic phosphorus reserves decrease over time in many agricultural soils, genetic manipulation of plants to enable secretion of potent phytases into the rhizosphere has been proposed as a promising approach to improve plant phosphorus nutrition. Several families of biotechnologically important phytases have been discovered and characterized, but little data are available on which phytase families can offer the most benefits toward improving plant phosphorus intake. We have developed transgenic *Arabidopsis thaliana* plants expressing bacterial phytases PaPhyC (HAP family of phytases) and 168phyA (BPP family) under the control of root-specific inducible promoter *Pht1;2*. The effects of each phytase expression on growth, morphology and inorganic phosphorus accumulation in plants grown on phytate hydroponically or in perlite as the only source of phosphorus were investigated. The most enzymatic activity for both phytases was detected in cell wall-bound fractions of roots, indicating that these enzymes were efficiently secreted. Expression of both bacterial phytases in roots improved plant growth on phytate and resulted in larger rosette leaf area and diameter, higher phosphorus content and increased shoot dry weight, implying that these plants were indeed capable of utilizing phytate as the source of phosphorus for growth and development. When grown on phytate the HAP-type phytase outperformed its BPP-type counterpart for plant biomass production, though this effect was only observed in hydroponic conditions and not in perlite. Furthermore, we found no evidence of adverse side effects of microbial phytase expression in *A. thaliana* on plant physiology and seed germination. Our data highlight important functional differences between these members of bacterial phytase families and indicate that future crop biotechnologies involving such enzymes will require a very careful evaluation of phytase source and activity. Overall, our data suggest feasibility of using bacterial phytases to improve plant growth in conditions of phosphorus deficiency and demonstrate that inducible expression of recombinant enzymes should be investigated further as a viable approach to plant biotechnology.

## Introduction

Phosphorus is an essential element required for plant growth and development. Most plants acquire phosphorus from soil in the form of inorganic phosphate, but soluble phosphate reserves in many agricultural soils rapidly decline due to highly demanding agricultural practices. As a consequence, crop production relies heavily on application of rock phosphate fertilizer to improve soil phosphorus availability. However, this approach is not sustainable on the long-term, and several recent analyses forecast that rock phosphate deposits worldwide will be largely depleted by the end of this century (Van Vuuren et al., 2010; Gaxiola et al., 2011). Furthermore, excessive application of phosphate fertilizer leads to soil pollution and eutrophication, and is also extremely inefficient as up to 80% of fertilizer is rapidly transformed into insoluble organic compounds or otherwise becomes inaccessible to plants (Holford, 1997; Schachtman et al., 1998; Gontia et al., 2012).

A large portion of soil phosphorus is present in different organic forms, including *myo*-inositol 1,2,3,4,5,6-hexakisphosphate (phytate) (Giles et al., 2011). Indeed, with its 6 phosphate groups covalently attached to 6 carbon atoms of *myo*-inositol ring, phytate is thought to be one of the most abundant forms of soil phosphorus reserves (Dalal, 1977; Turner et al., 2002). For example, phytate forms up to 25.3% of total extractable P in certain agricultural soils (Rugova et al., 2014). While very abundant in soil, phytate is not available to most plant species as their roots typically do not secrete to the rhizosphere sufficient quantities of phytate-degrading enzymes called phytases (Richardson et al., 2001; Mudge et al., 2003; Konietzny and Greiner, 2004). While plants do possess various types of phytases, they are largely intracellular or their expression is often restricted to a specific phase of plant development (Hegeman and Grabau, 2001; Richardson et al., 2001; Konietzny and Greiner, 2002; Greiner, 2007) and thus, cannot substantially contribute to improved plant growth in conditions of limited inorganic phosphate availability in soil.

In contrast to plants, many soil microorganisms, including various bacteria and fungi, synthesize and secrete highly abundant and active extracellular phytases (Mukhametzianova et al., 2012). These microbes are often used in agriculture as biofertilizers due to their collective ability to increase soil phosphate availability and to promote plant growth through the combined action of phytases, other secreted enzymes and exudates (Richardson and Simpson, 2011; Sharma et al., 2013; Souza et al., 2015). Many recent advances in plant biotechnology indicate that a promising transgenic technology could be developed to improve plant phosphorus nutrition by engineering plants to secrete microbial phytases (Secco et al., 2017). In principle, such approach can provide valuable benefits to crop productivity by generating additional pool of inorganic phosphate molecules derived from soil phytate (Fu et al., 2008), as well as offer an alternative strategy to help solve ecological problems of eutrophication by reducing both the amount of applied fertilizers and the degree of phytate accumulation in soil and water (Gontia et al., 2012; Yao et al., 2012).

Bacterial and eukaryotic phytases are typically classified into several major families based on important differences in structure, substrate specificity, pH-optimum and mechanism of phytate hydrolysis (Greiner, 2007; Lei et al., 2013). Based on pH-optimum of activity, all phytases are divided into alkaline and acid enzymes (Yao et al., 2012). Alkaline phytases feature a unique β-propeller fold (thus the name, BPP phytase enzymes) and a relatively high pH optimum of enzymatic activity in the pH range of 7–8 (Mullaney and Ullah, 2003, 2007; Jog et al., 2005; Balaban et al., 2016). In addition, BPP phytases are characterized by a very narrow substrate specificity geared uniquely toward phytate molecule (Mullaney and Ullah, 2003; Oh et al., 2004; Elkhalil et al., 2007). BPP phytases were originally discovered in the bacterial genus Bacillus but later also identified in other bacteria, as well as in fungi and plants (Greiner, 2007; Mullaney and Ullah, 2007). In contrast to alkaline phytases, which structurally form a very distinct group of enzymes, all acid phytases can be further subdivided into histidine acid phosphatases (HAPs), purple acid phosphatases (PAPs) and cysteine acid phytases (also known as protein tyrosine phosphatases or PTPs, mostly from ruminal bacteria), of which the HAP group features the vast majority of currently well-characterized phytases. HAPs are typically characterized by the presence of two pH-optima in the acid range and by a very broad substrate specificity: in addition to phytate they can hydrolyze various phosphorylated substrates, such as *p*-nitrophenyl phosphate (pNPP), AMP, ATP, fructose-1,6-bisphosphate, glucose-6-phosphate and other molecules (Oh et al., 2004; Greiner, 2007; Lei and Porres, 2007). In addition, all HAP family members harbor two highly conserved active site motifs, N-terminal RHGXRXP and C-terminal HD (Mullaney and Ullah, 2003; Bohm et al., 2010).

Several recent studies have reported generation and characterization of transgenic plants expressing microbial phytases from various families. Bacterial or fungal phytases have been expressed in transgenic tobacco, soybeans, alfalfa, corn, wheat, sweet potato, canola and *Arabidopsis thaliana* (Verwoerd et al., 1995; Richardson et al., 2001; Ullah et al., 2002; Brinch-Pedersen et al., 2003; Mudge et al., 2003; Chiera et al., 2004; Hong et al., 2004, 2008; Hamada et al., 2005; Lung et al., 2005; Wang et al., 2007, 2013; Bilyeu et al., 2008; Chen et al., 2008; Shen et al., 2008; Li et al., 2009). Initially HAPs have been widely used as the primary source of phytases for expression in plants, which in many cases has indeed resulted in better plant growth on phytate medium and higher accumulation of inorganic phosphorus in plant tissues in laboratory conditions. For example, transgenic soy roots expressing *Aspergillus ficuum* histidine acid phytase (AfPhyA) displayed up to 6 and 3.5 fold higher catalytic activity and inorganic phosphate content than wild type control plants, respectively (Li et al., 2009). Similarly, transgenic *A. thaliana* plants growing on phytate as the only source of phosphorus showed improved growth associated with overexpression of *Aspergillus niger* histidine acid phytase gene *phyA* in roots (Mudge et al., 2003), while expression of *Aspergillus niger* phytase in wheat decreased phytate content in seeds by 86% and had a positive impact on transgenic wheat nutritional properties (Brinch-Pedersen et al., 2003). In addition, expression of *Aspergillus niger* phytase fused with carrot extensin signal peptide in *A. thaliana* resulted in recombinant phytase secretion into rhizosphere concomitant with 20-fold increase in rhizosphere phytase activity (Richardson et al., 2001).

More recently, however, BPPs from *Bacillus subtilis* strains have emerged as the alternative and highly promising source of phytases for plant genetic engineering. Besides having an entirely different mode of action, this type of enzymes offers the additional advantage of being specific toward phytate, and thus, potentially not having detrimental side effects toward other aspects of phosphorus metabolism inside plant cells (Yip et al., 2003; Lung et al., 2005). For instance, transgenic tobacco plants expressing phytase 168phyA from *B. subtilis* showed up to twofold increase in biomass, as well as higher number of flowers and fruits compared to the wild type when grown on phytate as the only source of phosphorus (Yip et al., 2003). Similarly, expression of *B. subtilis* 168phyA phytase in *A. thaliana* led to a higher shoot dry weight and an increase in phosphorus content by 100% compared to the wild type (Lung et al., 2005). Similar results have more recently been obtained with a related BPP phytase PHY-US417 expressed in *A. thaliana* (Belgaroui et al., 2014, 2016).

While very encouraging results in transgenic plant research have been described using both HAP and BPP phytases, it is currently still not clear which family of phytases offers the most benefits for phosphorus metabolism in genetically modified plants while simultaneously causing as few side effects as possible. Indeed, negative effects of transgenic phytase expression in plants have been reported. For example, tobacco seeds from transgenic lines expressing bacillar BPP-type phytase 168phyA were characterized by smaller seed size and lower germination rates than their wild type counterparts (Yip et al., 2003; Lung et al., 2005). Hence, more research is needed to directly compare expression of HAP and BPP phytase families under similar conditions in plants, evaluate side by side their relative effects toward improving plant growth on phytate-containing medium and assess any potential side effects on plant metabolism.

To directly compare the relative effects of HAP- and BPP-type recombinant phytases on plant phosphorus metabolism *in vivo*, we generated transgenic *Arabidopsis thaliana* plants expressing 168phyA (a BPP-type phytase from *Bacillus subtilis* reference strain 168) (Tye et al., 2002) and PaPhyC (a HAP-type phytase from *Pantoea agglomerans*) (Greiner, 2004) fused with carrot extensin leader peptide under the control of root-specific inducible *Pht1;2* promoter from *A. thaliana* (Mudge et al., 2002, 2003; Nussaume et al., 2011). We grew these and control plants on hydroponic and soilless perlite media containing Na_2_HPO_4_ (inorganic phosphate, Pi) or phytate (*myo*-inositol hexakisphosphate, IHP) as the only source of phosphorus and evaluated phytase activity in roots, shoot growth and morphology, dry weight and shoot phosphorus content. We demonstrate that transgenic plants with high levels of 168phyA and PaPhyC phytase activity were able to efficiently utilize phytate from perlite and hydroponic media resulting in larger rosette diameter and leaf area, and higher phosphorus content than in control plants grown in the same conditions. Moreover, we show that PaPhyC-expressing plants display higher shoot dry weight than plants expressing 168phyA phytase, but only in hydroponic medium, indicating that under these conditions a HAP-type enzyme outperformed its BPP-type counterpart for biomass accumulation. Aside from shoot dry weight, all transgenic phytase-expressing plants had similar morphological characteristics of rosette leaves and did not show any evidence of changes in seed germination rates. Taken together, our data indicate that while both phytases can improve plant growth on phytate, under certain conditions PaPhyC may provide additional benefits over 168phyA for faster biomass accumulation.

## Materials and Methods

### Transgene Construction

Coding regions of HAP-type phytase gene *paPhyC* from *Pantoea agglomerans* (ABD85282.1) (Greiner, 2004) and BPP-type phytase gene *168phyA* from *Bacillus subtilis* (CAB13871.1) (Tye et al., 2002) were codon-optimized for expression in *A. thaliana* using the Codon Adaptation Tool software^1^ (Grote et al., 2005). *paPhyC* and *168phyA* coding regions were chemically synthesized (GenScript United States Inc.) as in-frame 5′ fusions with the carrot extensin leader sequence (Richardson et al., 2001) for efficient protein secretion and 3′ fusions with 6x-His (CATCATCATCATCATCAT) and Strep-tag II (TGGTCTCATCCTCAATTTGAAAAG) sequences (Supplementary Figures S1, S2). BamHI and SpeI recognition sites were added on 5′ and 3′ ends of the synthetic constructs, respectively, to facilitate further cloning. Both constructs were placed under the control of the full-length *Pht1;2* promoter (-2000 to -1 bp relative to the start codon of *AtPht1;2* gene) (Mudge et al., 2003), which was amplified from *A. thaliana* genomic DNA using primers 5′-CTGCAGGATCACTATACAACTCTGCACT-3′ and 5′-GGATCCCTAAGCCTCTCTTGTCTTTCC-3′ (PstI and BamHI restriction sites are underlined). Both *Pht1;2::phytase* constructs were inserted into pCBK05 binary vector (Riha et al., 2002) by replacing the vector-specific CaMV35S promoter using PstI and SpeI restriction enzymes, and the derived vector was designated pCEV03 (Supplementary Figure S3). Empty vector containing only the full-length *Pht1;2* promoter (without the phytase constructs) was used as a negative “promoter only” control.

### Plant Transformation, Selection and Growth Procedures

All three pCEV03 constructs were introduced into the *Agrobacterium tumefaciens* GV3101 strain, which was used to transform wild type *Arabidopsis thaliana* plants (Columbia ecotype) by the modified *in planta* method (Bechtold and Pelletier, 1998). *A. thaliana* seeds were cold treated overnight at 4°C, placed in an environmental growth chamber (Panasonic MLR-352, Japan) and grown under a 16-h light/8-h dark photoperiod at 20°C. Primary transformants T1 were selected on 0.5 Murashige and Skoog basal medium supplemented with 25 mg/l of phosphinothricine (BASTA) (Crescent Chemical, United States). BASTA-resistant plant lines were grown for at least three generations (T1, T2, T3) to identify homozygous lines with a single T-DNA insertion site per genome, as judged by a 3:1 segregation ratio for BASTA resistance in T2 plants. Homozygous for the T-DNA insertions plant lines were established from T3 generation and used for further analysis. Reverse transcription PCR (RT-PCR) and Western blotting with His-tag antibody were used to verify bacterial phytase expression in the transgenic lines (see below).

The ability of wild type and transgenic plants to utilize different sources of phosphorus was first determined by growth in hydroponic system (Tocquin et al., 2003). Plants were grown for 25 days in 1 l round sterile containers with liquid “standard” nutrient solution (Tocquin et al., 2003) at pH 5.7. Plants were also supplied with different sources of phosphorus in the medium: Pi (800 μM Na_2_HPO_4_), phytate (133 μM Na-IHP, Sigma–Aldrich) or grown with no added phosphorus source (No-P control). At least three biological replicates (7–12 plants each) per treatment per plant line were analyzed for various morphological and biochemical parameters.

For growth on perlite, seeds were germinated on sterile plates with 0.5 MS agar in a plant growth chamber (20°C day/20°C night, 16/8 h light/dark conditions) and grown until 4-leaf stage. Plants were then transferred to 6 pots (6 × 6.75 × 6 cm, W/L/H, 3 plants per pot) filled with 100 g of Perlite (Agroperlit, Vita Line) pre-fertilized with liquid “standard” nutrient solution (Tocquin et al., 2003) pH 5.0 ± 0.5 containing sodium phytate (133 μM Na-IHP, Sigma–Aldrich) as the only source of phosphorus. Plants were grown for 21 days in 20°C, 16/8 h light/dark conditions with regular fertilization by phytate-containing “standard” nutrient solution 2–3 times a week. At the end of the experiment, three to six independent replicates (the average of 3 individual plants in each pot) were collected for wild type and every transgenic plant line and analyzed for growth, morphology parameters and phosphorus content.

### Semi-quantitative RT-PCR and Western Blotting

RNA was extracted from roots grown in hydroponic conditions in the presence of phytate using the TRI reagent solution (Sigma). First strand cDNA synthesis from 1 μg of total RNA was conducted using RevertAid H Minus First Strand cDNA Synthesis Kit (Thermo Fisher Scientific) and Oligo-dT primer. The gene-specific PCR step was performed using the forward primer 5′-ATCACAACCACCACCTCCCTC-3′ specific to the first exon of both constructs (located inside the 2 kb *Pht1;2* promoter region) and reverse primers 5′-TCCATAGCCTTCAAAGCTTG-3′ or 5′-CCTTATCTCCATCAATAGCA-3′ specific to the codon-optimized sequences of *paPhyC* and *168phyA*, respectively.

Expression of 168phyA and PaPhyC phytase proteins in plants was confirmed by Western blot analysis. Plants were grown in hydroponic nutrient solution supplied with phytate for 28 days. Plant roots were collected, ground in liquid N_2_ with mortar and pestle and transferred to Protein Extraction Buffer (15 mM MES/Ca buffer containing 1 mM cysteine and 1 mM EDTA). Homogenized tissue was then spun at 12000 g for 10 min at 4°C. Supernatant was collected as soluble protein extract and stored in -20°C. Pellet was resuspended in cell wall extraction buffer [15 mM MES (pH 8.5), 1 mM EDTA, 100 mM NaCl, 1% Triton X-100, 0.5 mM CaCl2, 1 mM PMSF, 5 mM cysteine] and spun at 15,000 g for 10 min at 4°C. Supernatant was collected as cell wall protein extract fraction and stored at -20°C. For Western blotting 30 μg of each protein extract was incubated for 5 min at 85°C and subjected to SDS-PAGE (12.5% acrylamide gel). Separated proteins were transferred onto a PVDF membrane for 50 min at 90 V using Mini Trans-Blot Electrophoretic Transfer Cell (Bio-Rad). Membrane was blocked with 5% Skim Milk (Sigma–Aldrich) in PBS-T buffer for 2 h at RT with shaking. PVDF membrane was then incubated with primary antibodies (6x-His Epitope Tag Monoclonal Antibody HIS.H8, Thermo Fisher Scientific) at 1:3,000 dilution for 1 h at RT with shaking. The membrane was washed for 10 min three times in PBS-T buffer and incubated with secondary antibodies [Pierce Goat Anti-Mouse IgG, (H + L), Peroxidase conjugated, Thermo Fisher Scientific] at 1:10,000 dilution for 30 min. After washing in PBS-T and PBS three and two times, respectively, the membrane was visualized using a chromogenic substrate containing SuperSignal West Pico Stable Peroxidase Solution and SuperSignal West Pico Luminol/Enhancer Solution (Thermo Fisher Scientific).

### Phytase Activity Assays

Protein concentration was calculated with a Dc Protein Assay (Bio-Rad) using bovine serum albumin as a standard. Phytase activity was assayed by measuring the amount of released inorganic phosphate (Pi) with a modified ammonium molybdate method (Jog et al., 2005). Briefly, 50 μl of enzyme solution was added to 200 μl of 15 mM MES buffer pH 5.5 with 1.25 mM Na-IHP, 0.5 mM CaCl_2_ and incubated at 37°C for 1 h. The reaction was stopped by the addition of 50 μl of 50% TCA. Pellet was spun down for 5 min at 15,000 rpm. Blank controls were prepared by adding 50% TCA prior to the addition of enzyme. Colorimetric reactions with ammonium molybdate solution were then performed as previously described (Jog et al., 2005). Optical density was measured at 820 nm on a model 2550 Microplate Reader (Bio-Rad, United States). A calibration curve was built using concentrations of inorganic phosphate in the range of 5.625–90 μM. One unit (U) of phytase activity was defined as the amount of enzyme necessary to produce 1 μmol of inorganic phosphate per min at 37°C.

### Dry Weight and Total Phosphorus Content Measurement

For dry weight evaluation shoots of each individual plant were collected separately and washed with dH_2_O. Excess water was removed by lightly pressing plants on filter paper. Samples were dried in dry heat oven at 70°C for 48 h and dry weight of each shoot sample was measured on analytical balances. Phosphorus content in plant tissues was estimated by ashing dried plant material at 420°C for 4 h to overnight in a muffle furnace. Ash was then dissolved in 0.9 M H_2_SO_4_ (w/v, 10 mg of dried tissue/ 1 ml of 0.9 M H_2_SO_4_) for 24 h in RT. An aliquot of the supernatant was carefully taken and the total phosphorus content was measured by reaction with ammonium molybdate as described for phytase activity assay (Jog et al., 2005). Absorbance at 820 nm was measured and the inorganic phosphate concentration was determined from a calibration curve using KH_2_PO_4_ as the standard.

### Plant Morphology and Seed Germination Analysis

Each individual plant was harvested, washed with water and slightly dried with filter paper. Rosettes were gently separated. Plant material was placed on gel documentation system tray and straightened by tweezers. Shoot images were captured using E-box VX2 gel documentation system (VilberLourmat, Germany) with 1.4 MP Sony camera. After taking pictures, morphological parameters of plants (rosette diameter, total area of rosette leaves) were calculated using ImageJ software^2^. Rosette diameter was measured as the mean maximum distance between distal ends of two oppositely positioned rosette leaves. Calculated areas of all rosette leaves in a plant were added up to obtain values for total rosette leaf area. For seed germination analysis several hundred seeds from each line were germinated on sterile plates with 0.5 MS agar for 12 days in a growth chamber (22°C day/20°C night, 14 h light). Only seedlings that formed true first leaves were counted as germinated seeds.

### Statistical Analysis

Statistical significance for hydroponics-grown plants was determined using two-way ANOVA with Bonferroni’s correction for multiple comparisons. Data from perlite-grown plants were analyzed by one-way ANOVA with Bonferroni’s correction for multiple comparisons. Significance was set at *p* < 0.05. All analyses were performed on 3–6 biological replicates using GraphPad Prism version 7.04. Fold change estimates were obtained by dividing the values for transgenic plants by the average of all control A-line values for the same experimental condition.

## Results

### Generation of Transgenic Plants Expressing Bacterial PaPhyC and 168phyA Phytases

To test the effects of HAP and BPP phytase expression in *A. thaliana* on plant morphology and physiology, the coding regions of phytase genes *paPhyC* from *Pantoea agglomerans* and *168phyA* from *Bacillus subtilis* were codon-optimized for expression in plants (Chuluuntsetseg et al., 2015; Valeeva et al., 2015), fused with the carrot extensin leader sequence (Supplementary Figures S1, S2) and placed under the control of the full 2 kb inducible root-specific *Pht1;2* promoter from *A. thaliana* (Mudge et al., 2002, 2003). *A. thaliana* wild type plants (Columbia ecotype) were transformed with T-DNA containing these constructs (designated K-line for *168phyA* and G-line for *paPhyC*) (Supplementary Figure S3) and with a control construct harboring the full *Pht1;2* promoter but no phytase gene (designated A-line). Transformed lines harboring a single T-DNA insertion site per genome were identified by analyzing segregation ratios of BASTA resistance in T2 and T3 generations. Phytase mRNA expression was detected by RT-PCR (Supplementary Figure S4A) (Nyamsuren et al., 2015), and phytase protein expression was confirmed by Western blotting (Supplementary Figures S4B,C). Confirmed transgenic lines and control A-lines (harboring just the *Pht1;2* promoter) were selected for further morphological and physiological analyses.

### Roots of Transgenic Plants Show Elevated Levels of Phytase Activity

We first determined the levels of soluble and cell wall-associated phytase activity in roots of transgenic and control (wild type and A-lines) Arabidopsis plants. All plants were grown in hydroponic conditions with inorganic phosphate (Pi) or phytate (IHP) as the sole source of P for 25 days. Plant growth in the absence of any source of phosphorus in the medium (No-P control) was also attempted, but resulted in immediate developmental abnormalities and plant death by day 10, obstructing further long-term analysis of plants in this condition.

#### Soluble Phytase Activity

**FIGURE 1:**
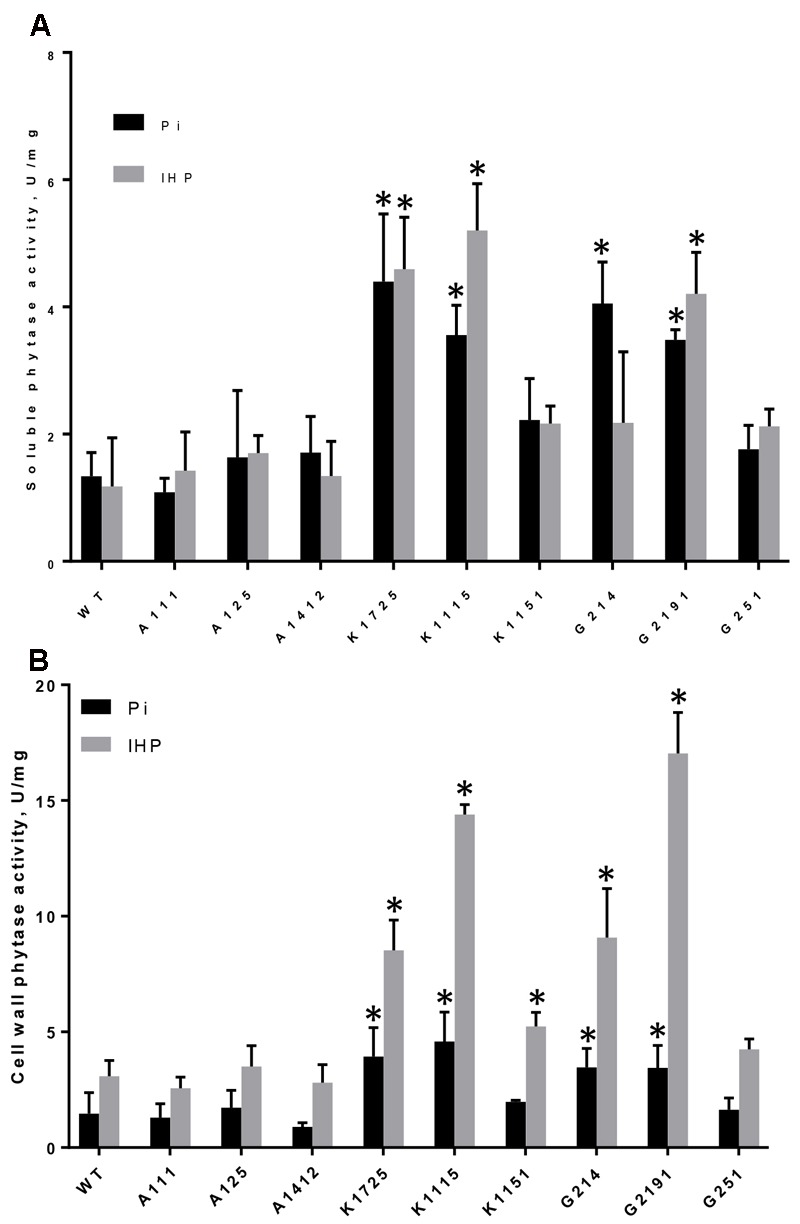
Phytase activity in roots of control and transgenic plants grown on Pi or IHP. **(A)** Phytase activity in soluble protein extracts from roots, **(B)** Phytase activity in root cell wall-bound protein extracts. Results are shown as mean ± SD for 3–6 replicates, with each replicate being root systems from 7 to 12 plants grown in one hydroponics container. WT, wild type roots; A111, A125, A1412 – roots of negative control lines; K1725, K1115, K1151 – roots from 168phyA -expressing plant lines; G214, G2191, G251 – roots from PaPhyC-expressing plant lines. ^∗^Indicates significance (*p* < 0.05) in two-way ANOVA multiple comparisons test. Comparison was made to the average of 3 control A-lines grown on either Pi or IHP.

Phytase activity in soluble root protein fractions îf wild type and plants of 3 control A-lines was not significantly different between Pi and phytate-supplied media and varied between 1.18–1.34 and 1.09–1.71 U/mg protein, respectively (**Figure 1A**). In contrast, phytase activity in soluble root protein extracts of transgenic plants from most K- and G-lines grown on both inorganic phosphorus and phytate was substantially higher than wild type and control levels (**Figure 1A**). Importantly, soluble root phytase activity on the medium with phytate was significantly higher in K1725, K1115 and G2191 transgenic plants (4.60, 5.20, and 4.21 U/mg, respectively) and also somewhat higher than in control plants in K1151, G214 and G251 lines (2.17, 2.18, and 2.12 U/mg, respectively) (**Figure 1A**). Higher levels of phytase activity observed in K- and G-line plants further confirm that transgenic PaPhyC and 168phyA phytases are expressed as enzymatically active proteins in plants.

#### Cell Wall-Associated Phytase Activity

Although high levels of phytase activity were detected in soluble intracellular fractions of transgenic roots on both Pi and phytate media, due to the presence of carrot extensin leader sequence in transgenic constructs we expected that the majority of enzymatically active PaPhyC and 168phyA proteins would be secreted outside plant cells. Since phytase activity in liquid hydroponic growth medium was below detection limits, we measured phytase activity in cell wall-associated protein fractions. Similar to the situation with soluble root protein extracts, cell wall-bound phytase activity in plants of most transgenic K- and G-lines grown on Pi was substantially higher than in wild type and control A-lines (**Figure 1B**).

Phytase activity in all K-line and G-line plants increased several fold when these plants were grown on phytate over levels observed in the same plants in Pi conditions (**Figure 1B**). Most importantly, all transgenic plants of K-lines grown on phytate showed variable, but significantly higher cell wall-bound phytase activities (between 1.77 and 4.86-fold increase) than control A-line plants grown in the same conditions (**Figure 1B**). Similarly, transgenic plants of G214 and G2191 lines grown on phytate showed significantly higher cell wall-bound phytase activities (3.07 and 5.75-fold increase, respectively) than control A-line plants grown in the same conditions. Cell wall-bound phytase activity levels in G251 line plants also showed 1.44-fold increase over average levels in control plants, though this number was not statistically significant (**Figure 1B**). Taken together, these data provide important evidence that activity of both transgenic phytases is mostly associated with cell wall, suggesting that they are indeed largely secreted.

### The Effects of Bacterial Phytase Expression on Shoot Growth and Morphology of Plants Grown in Hydroponic Conditions

To test the impact of bacterial phytase expression on plant morphology and growth characteristics, we evaluated wild type and transgenic shoots grown in hydroponics for the following parameters: total rosette leaf area and rosette diameter, shoot dry weight and overall phosphorus content.

#### Total Rosette Leaf Area and Diameter

**FIGURE 2:**
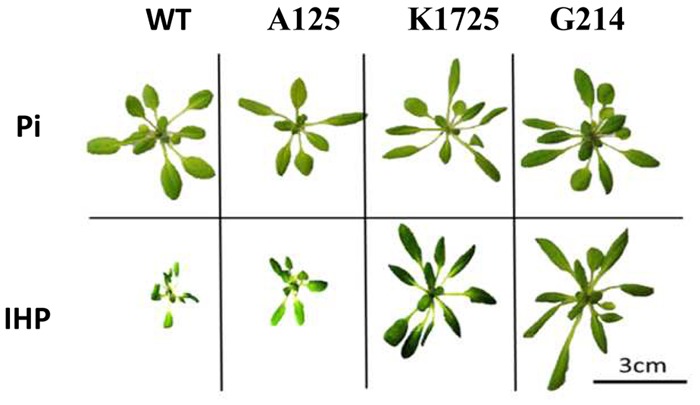
Morphology of wild type and transgenic plants grown in hydroponic conditions with inorganic phosphate Na_2_HPO_4_ (Pi) or phytate (IHP) as the sole source of phosphorus. Plants were grown in hydroponic conditions for 25 days. Representative pictures of individual wild type *A. thaliana* (WT), control A125 line (transgenic construct without phytase gene), transgenic plants of K1725 line (expressing 168phyA phytase) and G214 line (expressing PaPhyC phytase) are shown.

When grown in the presence of Pi, wild type and transgenic plants did not show any clear morphological differences (**Figure 2**, top panel and Supplementary Figure S5, top panel). Overall, rosette diameter ranged from 3.25 cm (line A125) to 4.08 cm (WT) to 6.18 cm (line K1151), while total rosette leaf area varied from 3.59 cm^2^ (line A125) to 5.49 cm^2^ (WT) to 7.12 cm^2^ (line K1151) (**Table 1**). In contrast, when grown on phytate, transgenic and control plants clearly displayed substantial morphological differences in overall plant size (**Figure 2**, bottom panel and Supplementary Figure S5, bottom panel). Specifically, rosette diameter decreased by 2.98-fold in wild type and on average by 3.38-fold in control A-line plants, compared to the same plants grown on Pi. However, 4 out of 6 plant lines expressing either transgenic phytase construct grew much better on phytate medium than their wild type and negative control transgenic counterparts. Specifically, K1725 and K1115 lines had 2.79 and 2.18 times larger rosette diameter, respectively, than the average A-line plant, while G214 and G2191 plants had rosette diameters larger than in A-lines by 2.64 and 2.15-fold, respectively. Only lines K1151 and G251 with the least amount of secreted phytase activity (**Figure 1B**) did not display any statistically significant improvement in rosette diameter over the negative control A-line plants when grown on phytate (**Table 1**).

**Table 1:** Shoot characteristics and total P content of wild type and transgenic plants grown on different sources of phosphorus in hydroponics.

Parameter	Treatment	WT	A111	A125	A1412	K1725	K1115	K1151	G214	G2191	G251
Rosette diameter, cm	Pi	4.08 ± 0.73	5.12 ± 0.17	3.25 ± 0.44	4.83 ± 0.71	3.94 ± 0.37	3.43 ± 0.12	6.18 ± 0.42	4.52 ± 0.30	4.62 ± 0.04	5.72 ± 0.16
						(0.89)	(0.78)	(1.40)	(1.03)	(1.05)	(1.30)
	IHP	1.37 ± 0.15	1.26 ± 0.03	1.12 ± 0.13	1.51 ± 0.34	3.63 ± 0.16^∗^	2.83 ± 0.15^∗^	1.55 ± 0.16	3.43 ± 0.50^∗^	2.79 ± 0.24^∗^	1.42 ± 0.07
						(2.79)	(2.18)	(1.19)	(2.64)	(2.15)	(1.09)
Rosette leaf area, cm^2^	Pi	5.49 ± 0.52	5.39 ± 0.80	3.59 ± 0.47	5.02 ± 0.49	4.71 ± 0.36	4.51 ± 0.53	7.12 ± 1.13	6.62 ± 1.43	5.52 ± 1.24	5.54 ± 0.28
						(1.01)	(0.97)	(1.52)	(1.42)	(1.18)	(1.19)
	IHP	0.88 ± 0.27	0.72 ± 0.15	1.59 ± 0.29^b^	0.87 ± 0.14	4.78 ± 1.30^∗^	2.65 ± 0.32^∗^	0.94 ± 0.13	5.41 ± 1.63^∗^	4.40 ± 1.39^∗^	0.71 ± 0.09
						(4.51)	(2.50)	(0.89)	(5.10)	(4.15)	(0.67)
Total P content, μg/plant	Pi	4.64 ± 0.60	4.47 ± 0.59	4.41 ± 0.23	4.38 ± 0.50	5.07 ± 0.26	4.59 ± 0.31	4.47 ± 0.44	4.92 ± 0.26	4.48 ± 0.58	4.43 ± 0.39
						(1.13)	(1.02)	(0.99)	(1.09)	(1.00)	(0.98)
	IHP	0.52 ± 0.04	0.44 ± 0.07	0.35 ± 0.14	0.44 ± 0.15	0.78 ± 0.16^∗^	0.68 ± 0.09^∗^	0.45 ± 0.08	1.56 ± 0.17^∗^	0.70 ± 0.03^∗^	0.33 ± 0.08
						(1.90)	(1.65)	(1.10)	(3.80)	(1.71)	(0.80)

*Results are shown as mean ± SD for 3–6 replicates, with each replicate being shoots from 7 to 12 plants grown in one hydroponics container. ^∗^Indicates significance (p < 0.05) in two-way ANOVA multiple comparisons test. Comparison was made to control A-lines grown on either Pi or IHP. Numbers in parentheses indicate fold change over the average of three control A-lines.*

As expected from visual analysis (**Figure 2** and Supplementary Figure S5), total rosette leaf area of wild type and control A-line plants was markedly reduced when grown on phytate compared to the same plants grown on Pi (**Table 1**). In contrast, transgenic K1725, K1115, G214 and G2191 plants grown on phytate had much larger rosette leaf area than their control counterparts, with only 0.88 cm^2^/plant and 1.06 cm^2^/plant for wild type and average A-line plants versus 4.78, 2.65, 5.41, and 4.40 cm^2^/plant for K1725, K1115, G214 and G2191 lines, respectively (**Table 1**). Similar to the situation with rosette diameter, K1151 and G251 plants did not display any significant improvement in rosette leaf area over the negative control A-line plants when grown on phytate (**Table 1**), likely reflecting insufficient amounts of secreted phytase activity. Overall, larger rosette diameter and rosette leaf area in most transgenic phytase-expressing lines grown on phytate clearly indicate that higher expression of 168phyA and PaPhyC phytases results in improved plant growth, likely due to their ability to extract phosphorus from phytate.

#### Shoot Dry Weight

**FIGURE 3:**
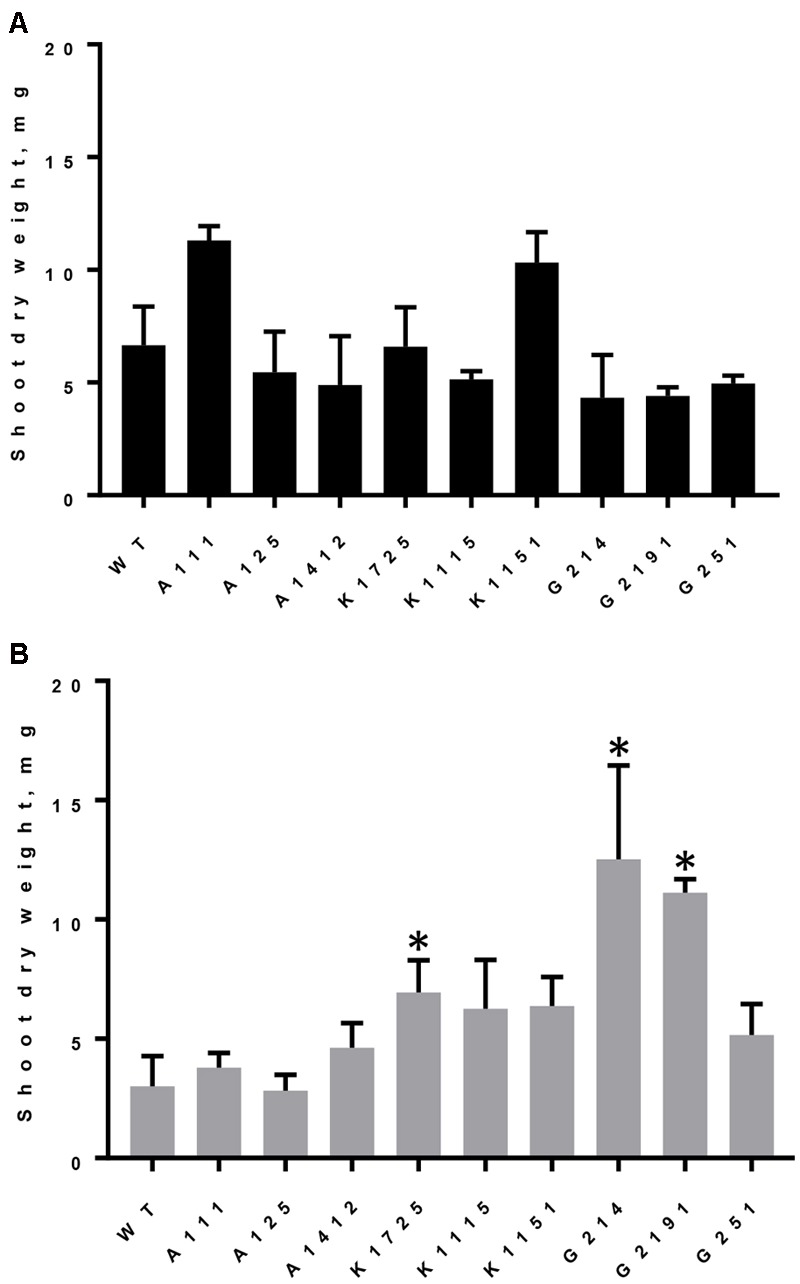
Dry weight accumulation in wild type and transgenic plants grown on different sources of phosphorus. Results are shown as mean ± SD for 3–6 replicates, with each replicate being shoots from 7 to 12 plants grown in one hydroponics container. **(A)** Shoot dry weight of plants grown on Pi, **(B)** Shoot dry weight of plants grown on phytate. WT, wild type plants; A111, A125, A1412 – plants of negative control lines; Transgenic plants of K- lines express 168phyA phytase and plants of G-lines express PaPhyC phytase. ^∗^Indicates significance (*p* < 0.05) in two-way ANOVA multiple comparisons test. Comparison was made to the average of 3 control A-lines grown on either Pi or IHP.

All wild type and transgenic plants produced large quantities of biomass, measured as shoot dry weight, when they were grown on Pi (**Figure 3A**). However, similar to the situation with total rosette leaf area and diameter, we observed significant differences between control and phytase-expressing transgenic plants grown on phytate. As expected for growth on phytate, dry weight of wild type and A-line shoots was reduced by 2.21 and on average by 1.93-fold, respectively, compared to growth of the same plants on Pi. On the other hand, when grown on phytate, shoot dry weight of all three K-lines was 1.68–1.86 times higher than shoot dry weight of A-line plants on the same medium (**Figure 3B**). Under the same conditions, shoot dry weight of G214 and G2191 plants expressing PaPhyC phytase was even higher and showed 3.35 and 2.98-fold increase over shoot dry weight of control A-line plants. Shoot dry weight of G251 plants, which harbor the least amount of secreted phytase activity, was only moderately higher (1.38-fold increase) than that of the average control A-line plants.

Interestingly, two-way analysis of variance with Bonferroni’s correction for multiple comparisons test indicates that when grown on phytate in hydroponics, plants of G-lines displayed statistically significant (*p* < 0.0007) differences in shoot dry weight accumulation as compared to all three K-lines. Specifically, shoot dry weight of G214 and G2191 plants (harboring *paPhyC* gene) was up to two times higher than that of K1725, K1115 and K1151 plants (harboring *168phyA*) (**Figure 3B**). Assuming that the rather minimal improvement in dry weight accumulation in the G251 line over control lines can be attributed to insufficient phytase expression, the data for the other two G-lines may support important functional differences between specific expression outcomes of these two genes in plants and suggest that under conditions tested (growth on phytate in hydroponics) high activity levels of PaPhyC (a HAP-type phytase) may promote higher biomass accumulation than 168phyA phytase (a BPP-type enzyme).

### The Effect of Bacterial Phytase Expression in Hydroponics-Grown Plants on Shoot Phosphorus Content

Wild type *A. thaliana* plants are largely unable to utilize phosphorus from phytate, and their overall inorganic phosphorus content decreases when plants are grown on phytate as the sole source of phosphorus as compared to Pi (Richardson et al., 2001). To test if transgenic plants expressing 168phyA and PaPhyC phytases are indeed scavenging more phosphorus from phytate than control plants, we measured internal phosphorus content in transgenic and control shoots. When all transgenic and control lines were grown in the presence of Pi, we observed no statistically significant difference in total Pi accumulation in the shoots, which varied from 4.38 to 5.07 μg/plant (**Table 1**). As expected from previous reports (Richardson et al., 2001; George et al., 2004), total Pi content in wild type and control A-lines grown on phytate decreased substantially (**Table 1**). While total Pi content in transgenic K- and G-lines grown on phytate also decreased, most transgenic plants expressing high levels of bacterial phytases maintained higher Pi levels as compared to control A-lines. Specifically, phosphorus content in transgenic plants increased over A-lines by 1.90-fold (K1725 line), 1.65-fold (K1115 line), 3.80-fold (G214 line), and 1.71-fold (line G2191) (**Table 1**). Phosphorus content in transgenic plants with lower levels of phytase activity (K1151 and G251) did not significantly differ from control plants. Overall, these data indicate that, when grown in hydroponic conditions, transgenic plants with high levels of recombinant PaPhyC or 168phyA phytase activity not only display improved growth in the medium with phytate, but do so by accumulating higher net phosphorus content than wild type or corresponding control plants.

### The Effects of Bacterial Phytase Expression on Plants Grown in Perlite

To test if expression of bacterial BPP and HAP phytases results in improved plant growth in soil-like conditions, we grew all experimental and control plants in pots containing soilless perlite medium. Unlike other soil-like compounds (i.e., vermiculate), perlite does not release its own nutrients and thus offers a superior choice for manipulating nutrient composition in the growth medium while simultaneously serving as an excellent solid substrate for root development. Hence, perlite is often used as a potting medium in experiments evaluating the effects of phosphorus uptake on plant development (Cabello et al., 2005; Garces-Ruiz et al., 2017). Plants were grown on perlite fertilized with “standard” nutrient solution at pH 5.0 ± 0.5, as higher pH values at 5.7 and above apparently resulted in the formation of insoluble phytate-containing precipitates, as previously reported for sand-vermiculate mixtures (Hayes et al., 2000). After 21 days of growth all control and transgenic plants were evaluated for shoot dry weight, rosette leaf diameter, total leaf area and total phosphorus content. Shoot dry weight of wild type and control plants of A-lines grown on phytate remained within 2.60–6.29 mg/plant range (**Table 2**). Overall, 3 K- and G-lines grown in perlite on phytate showed significant improvement in dry weight accumulation over wild type or A-line controls (**Table 2**). Specifically, K1725, K1115 and G2191 lines showed statistically significant increase in shoot dry weight (2.30-, 3.87-, and 3.29-fold increase, respectively), while the other 3 transgenic lines did not substantially differ from wild type and A-line controls. Importantly, the better-performing transgenic lines K1725, K1115 and G2191 are also characterized by the highest levels of transgenic phytase activity when grown on phytate (**Figure 1B**). This observation further highlights the apparent functional link between efficient phytase expression and growth improvement in conditions when phytate is the only source of phosphorus in the medium.

**Table 2:** Shoot characteristics, dry weight and total P content of wild type and transgenic plants grown on Perlite with phytate as the only source of phosphorus.

Parameter	WT	A111	A125	A1412	K1725	K1115	K1151	G214	G2191	G251
Shoot dry weight, mg	6.29 ± 1.94	2.60 ± 0.46^ns^	5.91 ± 2.16	3.78 ± 0.86	9.43 ± 2.84^∗^	15.87 ± 3.52^∗^	6.13 ± 1.32	6.16 ± 0.67	13.48 ± 1.80^∗^	5.90 ± 1.38
					(2.30)	(3.87)	(1.50)	(1.50)	(3.29)	(1.44)
Rosette diameter, cm	2.12 ± 0.46	2.72 ± 0.38	2.01 ± 0.50	1.89 ± 0.24	2.35 ± 0.34	3.56 ± 0.13^∗^	1.87 ± 0.37	1.60 ± 0.26	3.41 ± 0.43^∗^	1.60 ± 0.18
					(1.06)	(1.61)	(0.85)	(0.72)	(1.54)	(0.72)
Rosette leaf area, cm^2^	1.63 ± 0.39	1.93 ± 0.79	1.40 ± 0.51	1.85 ± 1.12	2.32 ± 0.70	4.83 ± 0.27^∗^	1.36 ± 0.43	0.94 ± 0.34	4.75 ± 1.30^∗^	0.99 ± 0.24
					(1.34)	(2.79)	(0.79)	(0.54)	(2.74)	(0.57)
Total P content, μg/plant	0.48 ± 0.09	0.51 ± 0.07	0.33 ± 0.06	0.28 ± 0.08	0.65 ± 0.15^∗^	0.92 ± 0.19^∗^	0.57 ± 0.05^∗^	0.27 ± 0.05	0.92 ± 0.11^∗^	0.23 ± 0.03
					(1.76)	(2.49)	(1.54)	(0.73)	(2.49)	(0.62)

*Results are shown as mean ± SD for 3–6 replicates, with each replicate being shoots from 3 individual plants grown in each pot with perlite. ^∗^Indicates significance (p < 0.05) in one-way ANOVA multiple comparisons test. Comparison was made to control A-lines grown on IHP. Numbers in parentheses indicate fold change over the average of three control A-lines.*

We next looked at the rosette diameter and leaf area parameters (**Table 2**). As expected, rosette diameter and leaf area of wild type and control plants grown on phytate did not show substantial variation and ranged between 1.89–2.72 cm and 1.40–1.93 cm^2^, respectively. Among all 6 168phyA and PaPhyC expressing lines, only the two lines with the highest levels of secreted recombinant phytase activity (K1115, G2191) displayed statistically significant increase in rosette diameter and leaf area over values for wild type and control plants.

Finally, we measured total phosphate content in all plants grown on perlite. Total phosphate content of wild type and control A-line plants ranged between 0.28 and 0.51 μg/plant (**Table 2**). In contrast, plants of all K-lines harbored significantly higher levels of phosphate (1.54 to 2.49-fold increase over A-lines). In addition, plants of G2191 line also displayed a 2.49-fold enrichment in phosphate levels over control values. Altogether, our data indicate that although a substantial degree of variation in growth parameters is observed in transgenic plants grown in hydroponics and soil-like conditions, transgenic lines showing the highest phytase activity levels have the potential to outperform wild type and control plants in terms of biomass production and total plant phosphate content in all conditions tested.

### Effects of Bacterial Phytase Expression in Plants on Seed Germination

Previous reports have indicated that expression of bacterial phytase genes in plants may cause problems with seed viability (Yip et al., 2003; Lung et al., 2005). To check for any negative effects of 168phyA and PaPhyC expression on seed germination, seeds from control and transgenic lines with the highest levels of phytase activity were planted on 0.5 MS agar medium in Petri dishes and scored for successful germination. Germination rates varied between 97.77% (wild type) and 100% (G214) (Supplementary Table S1). Thus, we conclude that expression of either bacterial phytase in *A. thaliana* does not lead to any substantial negative impact on seed germination. Overall, our data indicate that expression of both 168phyA and PaPhyC phytases in plants results in higher soluble and cell wall associated phytase activity, improved shoot growth and elevated phosphorus uptake when plants are grown on phytate without negatively impacting seed germination.

## Discussion

In this study, we evaluated consequences of bacterial HAP and BPP phytase expression in plants under identical conditions and demonstrated that high expression of both enzymes can significantly improve growth and morphology of transgenic plants grown on phytate hydroponically. Transgenic lines with the highest levels of phytase activity also display improved biomass accumulation and phosphorus content when grown on soilless perlite medium. Additionally, we discovered that in hydroponic conditions plants expressing PaPhyC phytase accumulate more biomass than plants expressing 168phyA phytase. Neither enzyme appears to induce detrimental changes in plant physiology or seed germination rates. Overall, our findings provide further indication that expression of bacterial phytases in transgenic plants can provide an important route for engineering crops with better tolerance to conditions of low soil inorganic phosphorus content.

### Microbial Phytases Are an Efficient Tool for Improving Plant Phosphorus Metabolism

Commercial use of microbial phytases is being developed as a promising strategy aimed at increasing crop yield and controlling soil and water pollution. Indeed, much effort has been made to improve phosphorus plant nutrition by co-cultivating plants with bacterial producers of secreted phytases (so-called bio-fertilizers) or by direct addition of purified microbial phytases to soil (Hayes et al., 2000; Zhengao and Huayong, 2001; Idriss et al., 2002; Vessey, 2003; Fuentes-Ramírez and Caballero-Mellado, 2005). While in theory highly promising, this approach has a limited potential due to high costs of recombinant enzyme production and the need to optimize the intricate interactions between plants, bacteria and different soil types (Zimmermann et al., 2003; George et al., 2004). One potentially promising alternative to bio-fertilizers is the use of genetically engineered plants secreting phytases of microbial origin into rhizosphere. Indeed, many recent reports indicate that various plants expressing microbial phytases can efficiently utilize phytate when grown on synthetic media in laboratory conditions. Most of such phytases used in transgenic research are members of the HAP type of phytases (Pen et al., 1993; Verwoerd et al., 1995; Brinch-Pedersen et al., 2000; Ponstein et al., 2002; Hong et al., 2004; Bilyeu et al., 2008; Wang et al., 2013). In addition, a few studies reported successful use of BPP phytases (Yip et al., 2003; Lung et al., 2005; Chan et al., 2006; Belgaroui et al., 2014; Belgaroui et al., 2016) and PAP enzymes (Xiao et al., 2005; Ma et al., 2009, 2012). TTP phytases, which represent a relatively rare family of phytases found mostly in ruminant bacteria (Yanke et al., 1998), have also been utilized (Hong et al., 2004). Thus, although most phytase families have been used in plant biotechnology, it is currently still unclear which phytase type offers the most benefits to transgenic plants while simultaneously causing the fewest side effects to plant physiology and cellular metabolism.

To answer this question, expression of phytases from different enzyme families needs to be analyzed in parallel and under the exact same conditions in plants. Surprisingly, very few studies analyzed simultaneous expression of more than one phytase *in planta*. In two studies, expression of two plant-derived *MtPHY1* and *MtPAP1* genes (both purple acid phosphatases) in white clover and in alfalfa resulted in improved phosphorus acquisition and increased biomass when plants where supplied with organic phosphorus (Ma et al., 2009, 2012). Similarly, two HAP phytase genes (*phyA* from *Aspergillus niger* and *appA* from *Escherichia coli*) were overexpressed under identical conditions in *Brassica napus* and both resulted in improved soil organic phosphorus utilization (Wang et al., 2013). Finally, in one study expression of two different families of phytases (a cysteine acid phytase *SrPf6* from ruminant bacterium *Selenomonas ruminantium* and a HAP phytase *appA* from *E. coli*) in rice seeds resulted in no developmental or seed abnormalities, though growth on organic phosphorus was not evaluated (Hong et al., 2004). However, to the best of our knowledge, comparative *in planta* analysis of expression phenotypes of members of the two most biotechnologically important families of phytases, HAP and BPP, has previously not been described.

### Expression of PaPhyC and 168phyA Phytases Improves Shoot Morphology and Phosphorus Uptake in Transgenic Plants Grown on Phytate

In this work we have directly compared expression in plants of a HAP phytase from *Pantoea agglomerans* and a BPP phytase from *Bacillus subtilis* using the same promoter and identical growth conditions. Specifically, we tested whether expression of either bacterial phytase in *A. thaliana* leads to not only improved plant growth on phytate but also results in better inorganic phosphorus accumulation in the shoots. Because expression level of microbial proteins in plants may not be optimal owing to different codon usage preferences in plants and bacteria (Gouy and Gauter, 1982; Hamada et al., 2005), we have performed codon optimization of coding regions for both genes without changing their amino acid sequences prior to the introduction of transgenes into plant genomes. The choice of promoter represents one of the most important ways to optimize heterologous gene expression. While many root-specific promoters have been used in different studies to direct phytase expression (Mudge et al., 2003; Zimmermann et al., 2003; Xiao et al., 2005; Li et al., 2009), the inducible *Pht1;2* promoter is not only one of the most well-characterized, but has also been shown to respond very well to conditions of phosphorus starvation in *A. thaliana* (Mudge et al., 2002, 2003). Thus, to specifically express microbial phytases in plant roots, both *paPhyC* and *168PhyA* genes have been cloned under the control of the full 2 kb *A. thaliana* promoter *Pht1;2* (Mudge et al., 2003). To facilitate secretion of transgenic proteins into the rhizosphere, we fused the coding region of both phytases with carrot extensin leader sequence (Chen and Varner, 1985). To better characterize the influence of transgenic phytase expression on plant morphology and physiology, we turned to growing transgenic and control plants on hydroponics. This experimental set-up has allowed us to obtain large volume of plant root systems for protein expression analysis, as well as for morphological and biochemical assays. In parallel, we have evaluated transgenic plant performance in soilless perlite medium, which mimics more natural plant growth conditions and whose nutrient composition can be easily manipulated.

Our data indicate that transgenic plants of K1725, K1115, and K1151 lines (expressing 168phyA phytase) and of G214 and G2191 lines (expressing PaPhyC phytase) display much higher phytase activity levels in root extracts (especially in cell-wall bound fractions) on phytate medium as compared to wild type and empty vector control plants. These results further confirm that expression of both bacterial phytases, as expected, was not only induced under phosphate-starvation conditions but resulted in protein secretion across plasma membrane. In contrast, phytase activity in G251 line was not significantly induced, possibly highlighting important differences in specific T-DNA integration sites. Overall, our data correlate well with previously published analysis of transgenic *A. thaliana* plants expressing a HAP-type *Aspergillus niger* phytase under the control of the same *Pht1;2* promoter, which similarly had much higher activity on low Pi medium (Mudge et al., 2003).

Expression of bacterial phytases in plants has previously been associated with important changes in plant morphology, physiology and phosphorus content. For instance, expression of 168phyA phytase in tobacco and in *A. thaliana* resulted in increased shoot biomass (up to 1.7–2.2 fold) and 27–36% higher phosphorus content as compared to wild type (Yip et al., 2003; Lung et al., 2005). Here we show that expression of bacterial genes encoding a HAP phytase from *P. agglomerans* and a BPP-type phytase from *B. subtilis* resulted in improved plant growth on hydroponic medium with phytate as the sole source of phosphorus. Specifically, we observed increased rosette diameter (2.18–2.79 fold) and leaf area (2.50–5.10 fold), increased dry weight (1.86–3.35 fold) and significantly elevated shoot phosphorus content (1.65–1.90 fold), as compared to control plants, but only in transgenic plants with relatively high levels of phytase expression. Similarly, when transgenic plants with high levels of bacterial phytase expression (specifically, lines K1725, K1115, G2191 and for some parameters also lines K1151 and G214) were grown on perlite, we observed comparable levels of growth improvements, including up to 3.87-fold increase in shoot dry weight, up to 1.61-fold increase in rosette diameter, up to 2.79-fold increase in rosette leaf area and up to 2.49-fold increase in total P content. Taken together, our data clearly indicate that plants efficiently secreting both 168phyA and PaPhyC phytases are capable of cleaving phytate into inorganic phosphate and *myo*-inositol, leading to an increased overall inorganic phosphorus intake and improved growth.

Several lines of evidence support the notion that the presence of both transgenic phytases does not lead to any adverse effects on plant morphology and physiology. First, leaf morphology of all transgenic plants grown on Pi or phytate remained normal. Second, we observed no change in seed germination efficiency of transgenic plants. Third, the majority of phytase activity appears to be cell wall associated, suggesting that heterologous expression of bacterial phytases in these conditions is unlikely to cause any major physiological changes in the cytoplasm. We conclude that expression of bacterial phytases in *A. thaliana* leads to improved plant growth and phosphorus acquisition on hydroponic medium with phytate without any major detrimental outcomes. Thus, our results provide additional experimental support for the notion that the power of biotechnology can be harnessed to improve plant growth on marginal soils with little Pi but abundant phytate reserves.

### Comparison of PaPhyC and 168phyA Enzyme Potentials for Transgenic Plant Research

While physiological phenotypes of PaPhyC expression in plants have not been evaluated prior to this study, many other members of the HAP family of phytases have been extensively used to engineer both model plants and crops. In most cases, expression of a HAP-type phytase in plants led to important beneficial changes in plant morphology and metabolism, including better P accumulation and growth on phytate-containing medium (Gontia et al., 2012; Yao et al., 2012). 168phyA phytase has previously been expressed in tobacco and *A. thaliana*, though under different promoters (Yip et al., 2003; Lung et al., 2005; Chan et al., 2006). A closely related phytase from *B. subtilis* strain 417 (72% amino acid identity and 84% similarity to 168phyA phytase) has also recently been used for this purpose (Belgaroui et al., 2014, 2016). When grown on phytate, plants expressing these BPP-type phytases displayed twofold higher biomass levels (Belgaroui et al., 2016), as well as a significant increase in shoot P concentration compared to control plants (Lung et al., 2005; Belgaroui et al., 2016). However, in some cases these phenotypes were accompanied by negative effects on plant physiology, including smaller size and lower germination rates of transgenic tobacco seeds expressing 168phyA phytase (Yip et al., 2003; Lung et al., 2005). Thus, an important conclusion from our study is that expressing 168phyA phytase as a secreted protein under the control of inducible *Pht1;2* promoter does not cause any apparent adverse physiological effects and represents an effective route to utilize a BPP-type enzyme in applications of plant biotechnology.

Our data on 168phyA and PaPhyC expression in *A. thaliana* indicate that both phytases, when highly expressed under identical conditions and from the same promoter, clearly improved plant growth and P acquisition from phytate to a similar degree, both in hydroponics and perlite. Interestingly, the major difference between 168phyA and PaPhyC phytase expression in our experiments appears to be the phenotype of dry weight accumulation in conditions of hydroponics, when plants highly expressing PaPhyC are characterized by overall significantly higher shoot dry weight than their 168phyA-expressing counterparts. Taken at face value, this observation may suggest that a HAP-type phytase could be more beneficial when increased shoot biomass is the desired outcome of transgenic manipulations. However, when the same plants were grown on perlite with phytate as the only source of phosphorus, we did not detect any significant differences in shoot dry weight accumulation between 168phyA and PaPhyC expressing plants (though both transgenics clearly improved plant growth over control lines). This observation supports and correlates well with previous data indicating that growth improvement in laboratory conditions *in vitro* does not necessarily translate to growth improvement in more natural field conditions. One possible explanation for the observed differences in dry weight accumulation between 168phyA and PaPhyC expressing plants in hydroponics and perlite is that enzyme’s access to the substrate may be more limited in perlite than in hydroponic conditions. Indeed, in a hydroponic system roots can move more freely than when plants are grown in soil-like conditions, affecting access to the substrate and possibly even its local concentration. This potentially can help explain both our data and results of other research groups when excellent growth phenotypes of many transgenic plants grown in laboratory conditions are often compromised when these same plants are grown in soil (George et al., 2004, 2005), though some of these setbacks can apparently be modulated or even reversed through other considerations, such as a choice of promoter (Wang et al., 2009), phytase gene, signal sequences or soil types (Ma et al., 2012).

It should be noted that additional factors can influence the effects of transgenic phytase expression on plant growth. For example, some bacterial phytases expressed in plants are often post-translationally modified (Chan et al., 2006; Chen et al., 2008), raising the possibility that the observed differences in plant morphology may at least partially arise from distinct protein modification pathways affecting individual phytase molecules. Furthermore, individual enzyme’s preferences for optimal pH and temperature can have a major impact on phytase activity in plants. For example, PaPhyC phytase has a single pH optimum at pH 4.5 and temperature optimum around 60°C when purified from bacteria (Greiner, 2004). On the other hand, 168phyA phytase expressed in Bacillus preferred more neutral pH and similarly high optimal temperature (Tye et al., 2002). As pH of the hydroponic growth medium at the beginning of our experiments was set at 5.7, and pH of perlite medium was set even lower, these environmental parameters could have differentially affected recombinant PaPhyC and 168phyA enzymes, resulting in different outcomes. Finally, as tolerance to organic acids (specifically, citrate and malate) secreted by roots under phosphorus stress appears to be a determining factor affecting performance of at least some phytase enzymes in the rhizosphere (Tang et al., 2006; Lei et al., 2013), the ability of 168phyA and PaPhyC to tolerate these compounds can also have important implications. Nevertheless, our data provide the first glimpse into physiological differences between expressing individual members of HAP and BPP phytase families in plants and can serve as a useful guide into future research in this area.

Overall, while various biochemical constraints are clearly important and need to be taken into consideration when designing future plant biotechnologies, our data clearly demonstrate that for both phytases those transgenic lines that harbor the highest levels of recombinant phytase activity also consistently demonstrate greater growth improvement in both hydroponics and soil-like conditions. Thus, our data strongly reinforce the notion that the actual amount of enzyme activity is essential and can be a limiting factor in some transgenic studies. It is likely that while in hydroponic conditions the transgenic effects are more evident at a wider range of transgenic phytase activity levels, in a more real soil-like situations the amount of available active phytase needs to pass a certain threshold level to have a more significant impact on plant physiology. In general, while it is not uncommon for transgenic plant lines to display both different levels of phytase expression and different effects on plant growth in P-deficient conditions (George et al., 2004), our findings provide valuable quantitative comparative data on important correlations between higher phytase expression levels and greater improvements in growth phenotypes when plants are grown on phytate.

## Author Contributions

All authors have contributed significantly to this work and analyzed the data. MS and ES designed the experiments. LV and CN performed the experiments and collected the data. LV, MS, and ES wrote the article with contributions from all authors.

## Conflict of Interest Statement

The authors declare that the research was conducted in the absence of any commercial or financial relationships that could be construed as a potential conflict of interest.

## Footnotes

1 http://www.jcat.de/

2 http://imageJ.nih.gov
