# Supplementary material for: Heterologous Expression of Secreted Bacterial BPP and HAP Phytases in Plants Stimulates Arabidopsis thaliana Growth on Phytate
Source: Front Plant Sci. 2018 Feb 20;9:186. doi: 10.3389/fpls.2018.00186 (PMC5826191; doi:10.3389/fpls.2018.00186)
Supplement: Supplementary file 1 [file Presentation_1.PDF]

ATGGGAAGAATTGCTAGAGGATCTAAGATGTCTTCTTTGATTGTTTCTTTGTTGGT
TGTTTTGGTTTCTTTGAATTTGGCTTCTGAAACTACTGCTCAAGAAACTCCTGAAG
GATATCAATTGCAACAAGTTTTGATTATGTCTAGACATAATTTGAGAGCTCCTTT
GGCTAATAATGGATCTGTTTTGGAACAATCTACTCCTAATGAATGGCCTGAATGG
GATGTTCCTGGAGGACAATTGACTACTAAGGGAGGAGTTTTGGAAGTTTATATG
GGACATTATATGAGAGAATGGTTGGCTGAACAAGGAATGGTTAAGACTGGAGAA
TGTCCTGCTGCTGATTCTGTTTATGCTTATGCTAATTCTTTGCAAAGAACTGTTGC
TACTGCTCAATTTTTTATTACTGGAGCTTTTCCTGGATGTGATGTTCCTGTTCATC
ATCAAGAAAAGATGGGAACTATGGATCCTACTTTTAATCCTGTTATTACTGATAA
TTCTCCTGAATTTAGAGAACAAGCTTTGAAGGCTATGGAAACTGAAAGAAAGAA
GATGCAATTGACTGAATCTTATAAGTTGTTGGAAGAAATGACTAATTATGCTGAT
GTTCCTTCTTGTAAGGAAAAGAAGGATTATTCTTTGGCTGATGCTAAGGATACTT
TTTCTGCTGATTATGAAAAGGAACCTGGAGTTTCTGGACCTTTGAAGGTTGGAAA
TTCTTTGGTTGATGCTTTTACTTTGCAATATTATGAAGGATTTCCTGCTGATCAAG
TTGCTTGGGGAGAAATTAAGACTGATCAACAATGGAGAGTTTTGTCTAAGTTGA
AGAATGGATATCAAGATTCTTTGTTTACTTCTACTGAAGTTGCTCAAAATGTTGC
TAAGCCTTTGGTTAAGTATATTGATAAGACTTTGGTTACTGAACAAGCTAAGGCT
CCTAAGATTACTTTGTTGGTTGGACATGATTCTAATATTGCTTCTTTGTTGACTGC
TTTGGATTTTAAGCCTTATCAATTGCATGATCAACAAGAAAGAACTCCTATTGGA
GGAAAGATTGTTTTTCAAAGATGGCATGATAAGAATTCTAATCAAGAATTGATG
AAGATTGAATATGTTTATCAATCTTCTGAACAATTGAGAAATGCTTCTGTTTTGTC
TTTGCAATCTCCTGCTCAAAGAGTTACTTTGGAATTGAAGGGATGTCCTGTTGAT
GTTAATGGATTTTGTCCTGTTGATAAGTTTAATGCTGTTATGAATAATGCTGCTAA
GCATCATCATCATCATCATTGGTCTCATCCTCAATTTGAAAAGTAA

Supplemental Figure 1. Sequence of codon-optimized for expression in *A. thaliana paPhyC* coding region including carrot extensin leader sequence (green), 6xHis (purple) and Strep tagII (blue) regions. Start and stop codons are shown in red.

ATGGGAAGAATTGCTAGAGGATCTAAGATGTCTTCTTTGATTGTTTCTTTGTTGGT
TGTTTTGGTTTCTTTGAATTTGGCTTCTGAAACTACTGCTAAGGTTCCTAAGACTA
TGTTGTTGTCTACTGCTGCTGGATTGTTGTTGTCTTTGACTGCTACTTCTGTTTCTG
CTCATTATGTTAATGAAGAACATCATTTTAAGGTTACTGCTCATACTGAAACTGA
TCCTGTTGCTTCTGGAGATGATGCTGCTGATGATCCTGCTATTTGGGTTCATGAA
AAGCATCCTGAAAAGTCTAAGTTGATTACTACTAATAAGAAGTCTGGATTGGTTG
TTTATGATTTGGATGGAAAGCAATTGCATTCTTATGAATTTGGAAAGTTGAATAA
TGTTGATTTGAGATATGATTTTCCTTTGAATGGAGAAAAGATTGATATTGCTGCT
GCTTCTAATAGATCTGAAGGAAAGAATACTATTGAAGTTTATGCTATTGATGGAG
ATAAGGGAAAGTTGAAGTCTATTACTGATCCTAATCATCCTATTTCTACTAATAT
TTCTGAAGTTTATGGATTTTCTTTGTATCATTCTCAAAAGACTGGAGCTTTTTATG
CTTTGGTTACTGGAAAGCAAGGAGAATTTGAACAATATGAAATTGTTGATGGAG
GAAAGGGATATGTTACTGGAAAGAAGGTTAGAGAATTTAAGTTGAATTCTCAAA
CTGAAGGATTGGTTGCTGATGATGAATATGGAAATTTGTATATTGCTGAAGAAG
ATGAAGCTATTTGGAAGTTTAATGCTGAACCTGGAGGAGGATCTAAGGGACAAG
TTGTTGATAGAGCTACTGGAGATCATTTGACTGCTGATATTGAAGGATTGACTAT
TTATTATGCTCCTAATGGAAAGGGATATTTGATGGCTTCTTCTCAAGGAAATAAT
TCTTATGCTATGTATGAAAGACAAGGAAAGAATAGATATGTTGCTAATTTTGAAA
TTACTGATGGAGAAAAGATTGATGGAACTTCTGATACTGATGGAATTGATGTTTT
GGGATTTGGATTGGGACCTAAGTATCCTTATGGAATTTTTGTTGCTCAAGATGGA
GAAAATATTGATAATGGACAAGCTGTTAATCAAAATTTTAAGATTGTTTCTTGGG
AACAAATTGCTCAACATTTGGGAGAAATGCCTGATTTGCATAAGCAAGTTAATCC
TAGAAAGTTGAAGGATAGATCTGATGGACATCATCATCATCATCATTGGTCTCAT
CCTCAATTTGAAAAGTAA

Supplemental Figure 2. Sequence of codon-optimized for expression in *A. thaliana 168phyA* coding region including carrot extensin leader sequence (green), 6xHis (purple) and Strep tagII (blue) sequences. Start and stop codons are shown in red.

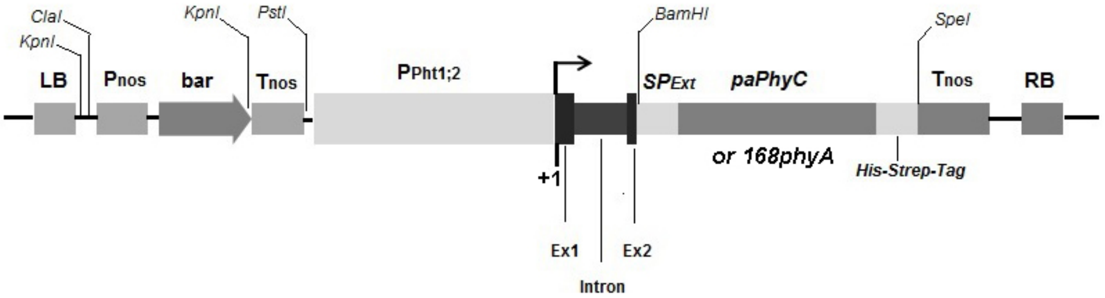

Supplemental Figure 3. Map of binary vector pCEV03 (~8.6 kb) for *paPhyC* and *168phyA* phytase gene expression in *A. thaliana* under the control of *Pht1;2* promoter. Pnos – nos promoter; bar – BASTA resistance gene; Tnos – nos terminator; LB and RB –left and right T-DNA borders; $P_{Pht1;2}$ – *Pht1;2* gene promoter (2 kb); +1 – transcription start site; Ex1, Ex2, Intron – first and second exon sequences and intron present in 5' untranslated region of *Pht1;2* promoter; SP_{Ext}- leader sequence of carrot extensin; *paPhyC* - *P. agglomerans* phytase coding region; *168phyA* – *B. subtilis* phytase coding region; His-Strep-Tag - sequence of 6xHis and Strep-tag II. Restriction sites are marked by curved lines.

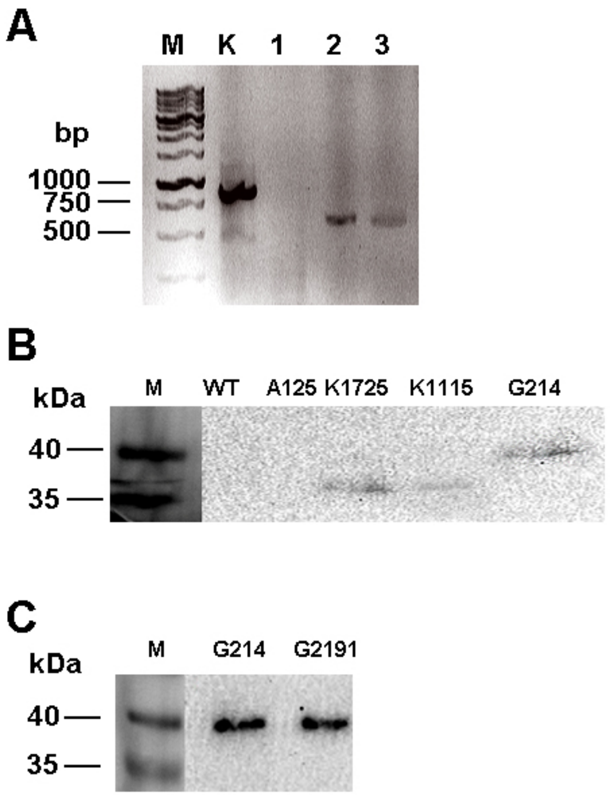

Supplemental Figure 4. RT-PCR and Westerm blot analysis of *paPhyC* and *168phyA* transgene expression in *A. thaliana*. (A) RT-PCR to detect mRNA expression of *paPhyC* transgene in *A. thaliana*. RNA was extracted from wild-type (lane 1), G214 (lane 2) and G2191 (lane 3) plants harboring the *paPhyC* transgene. To distinguish between true RT-PCR products and PCR products from potentially contaminating genomic DNA from T-DNA integration locus, primers were selected to amplify across the intron present in *Pht1;2* promoter (Mudge et al., 2003). A successful RT-PCR amplifies 630 bp PCR product from *paPhyC* mRNA, while PCR from genomic DNA produces 885 bp product. K - positive control PCR with genomic DNA from G214 line. M - molecular weight DNA markers. RT-PCR results for *168phyA* mRNA expression in *A. thaliana* (lines K1115 and K1725) have been published previously (Nyamsuren et al., 2015). **(B, C)** Western blot analysis of *A. thaliana*-expressed 168phyA and PaPhyC phytases. **(B)** Root protein extracts were prepared from wild type (WT) plants, control A125 line ("promoter only" transgenic construct), transgenic plants of K1725 and K1115 lines (expressing 168phyA phytase) and G214 line (expressing PaPhyC phytase). **(C)** Root protein extracts were prepared from G214 and G2191 lines expressing PaPhyC phytase.

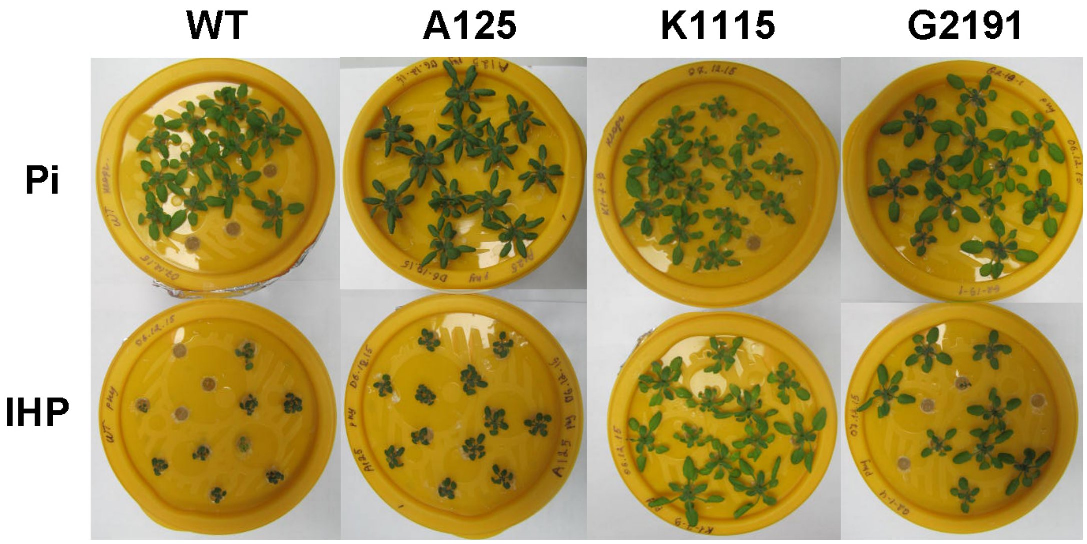

Supplementary Figure 5. Growth of wild type and transgenic plants in hydroponic conditions with inorganic phosphate Na_2HPO_4 (Pi) or phytate (IHP) as the sole source of phosphorus. Representative pictures (top-down view) of containers with wild type *A. thaliana* (WT), control A125 line (transgenic construct without phytase gene), transgenic plants of K1115 line (expressing 168phyA phytase) and G2191 line (expressing PaPhyC phytase) are shown.

Supplemental Table 1. Germination rates of wild type and transgenic *A. thaliana* seeds.

Parameter		WT	A125	K1725	K1115	G214	G2191
Total seed number		449	356	162	313	313	467
Germinated	Number of seeds	439	354	159	309	313	462
	%	97.77	99.44	98.15	98.72	100	98.93
Not germinated	Number of seeds	10	2	3	4	0	5
	%	2.23	0.56	1.85	1.28	0	1.07
